# Abscisic Acid Stimulates Glucagon-Like Peptide-1 Secretion from L-Cells and Its Oral Administration Increases Plasma Glucagon-Like Peptide-1 Levels in Rats

**DOI:** 10.1371/journal.pone.0140588

**Published:** 2015-10-21

**Authors:** Santina Bruzzone, Mirko Magnone, Elena Mannino, Giovanna Sociali, Laura Sturla, Chiara Fresia, Valeria Booz, Laura Emionite, Antonio De Flora, Elena Zocchi

**Affiliations:** 1 Department of Experimental Medicine (DIMES), Section of Biochemistry, and CEBR, University of Genova, Genova, Italy; 2 Animal facility, IRCCS AOU San Martino - IST Istituto Nazionale per la Ricerca sul Cancro, Genova, Italy; Consiglio Nazionale delle Ricerche, ITALY

## Abstract

In recent years, Abscisic Acid (ABA) has been demonstrated to be involved in the regulation of glucose homeostasis in mammals as an endogenous hormone, by stimulating both insulin release and peripheral glucose uptake. In addition, ABA is released by glucose- or GLP-1-stimulated β-pancreatic cells. Here we investigated whether ABA can stimulate GLP-1 release. The human enteroendocrine L cell line hNCI-H716 was used to explore whether ABA stimulates *in vitro* GLP-1 secretion and/or transcription. ABA induced GLP-1 release in hNCI-H716 cells, through a cAMP/PKA-dependent mechanism. ABA also enhanced GLP-1 transcription. In addition, oral administration of ABA significantly increased plasma GLP-1 and insulin levels in rats. In conclusion, ABA can stimulate GLP-1 release: this result and the previous observation that GLP-1 stimulates ABA release from β -cells, suggest a positive feed-back mechanism between ABA and GLP-1, regulating glucose homeostasis. Type 2 diabetes treatments targeting the GLP-1 axis by either inhibiting its rapid clearance by dipeptidyl-peptidase IV or using GLP-1 mimetics are currently used. Moreover, the development of treatments aimed at stimulating GLP-1 release from L cells has been considered as an alternative approach. Accordingly, our finding that ABA increases GLP-1 release *in vitro* and *in vivo* may suggest ABA and/or ABA analogs as potential anti-diabetic treatments.

## Introduction

Abscisic acid (ABA) is a phytohormone regulating fundamental physiological functions in plants [1, 2]. ABA is also an endogenous hormone in humans, regulating different cell responses and functions, including activation of innate immune cells and stimulation of insulin release and glucose uptake [3–6]. The signaling cascade of ABA in mammalian cells involves ABA binding to lanthionine synthetase C-like protein 2 (LANCL-2) and cAMP production [7–9]. Pro-inflammatory stimuli induce ABA production and release from human granulocytes, monocytes, keratinocytes and fibroblasts [3, 10–12] and ABA stimulates cell-specific functional activities in granulocytes (chemotaxis, phagocytosis, release of NO and reactive oxygen species), monocytes (chemotaxis, release of TNF-α, monocyte chemoattractant protein-1, metalloprotease 9 and prostaglandin E2), vascular smooth muscle cells (cell proliferation and migration), keratinocytes (release of NO, PGE2, and TNF-α) and fibroblasts (migration) [3, 10–12].

Several observations indicate that ABA is also involved in the regulation of glucose homeostasis in mammals as an endogenous hormone: i) ABA is released by human and murine pancreatic β-cells in response to high glucose, and nanomolar ABA triggers glucose-independent and potentiates glucose-dependent insulin secretion from these cells [4]; ii) oral glucose administration increases plasma ABA concentration ([ABA]_p_) in healthy human subjects [5]; iii) ABA stimulates glucose uptake by rodent adipocyte and myoblast cell lines [5]. In line with these data, Guri et al. observed that a chronic oral administration of exogenous ABA reduced the fasting plasma glucose concentration and ameliorated glucose tolerance in leptin receptor-deficient (db/db) mice [13].

Interestingly, the increase of [ABA]_p_ in response to an oral glucose load in healthy subjects was less consistently observed when the same subjects were administered glucose intravenously [5]. Oral, but not intravenous, glucose administration is followed by the release of the incretin glucagon-like peptide 1 (GLP-1), a gastrointestinal hormone secreted by enteroendocrine L-cells in response to nutrients, hormones and neurotransmitters. GLP-1 stimulates insulin and inhibits glucagon release, thereby contributing to the regulation of glycemia [14–16]. A possible explanation for the different effect of intravenously or orally administered glucose on [ABA]_p_ could come from the observation that GLP-1 stimulates ABA release by insulin-secreting cells, both in the presence of low- (2 mM) or of high- (25 mM) glucose concentrations [5].

In this study, we investigated whether ABA affects GLP-1 secretion by enteroendocrine cells, a process known to be regulated by the [cAMP]_i_ [14], thereby addressing the possible existence of a positive feed-back mechanism between ABA and GLP-1, regulating glucose homeostasis.

## Methods

### hNCI-H716 cell culture and GLP-1 secretion studies

The human L cell line hNCI-H716, derived from a poorly differentiated adenocarcinoma of the cecum, was obtained from the American Type Culture Collection (Manassas, VA). Cells were grown in suspension in RPMI-1640 (Sigma, Milano, Italy), supplemented with 10% fetal bovine serum (FBS), 50 U/ml penicillin and 50 μg/ml streptomycin.

For GLP-1 secretion assays, a protocol similar to the one described in [17] was followed: briefly, hNCI-H716 cells were seeded on Matrigel matrix (Becton Dickinson, Bedford, MA), at the density of 2x10^5^ cells/well in 24-well plates, in DMEM medium supplemented with 10% FCS, 50 U/ml penicillin, and 50 μg/ml streptomycin. After 48 h, cells were washed in Hank’s Balanced Salt Solution (HBSS) and then incubated for 2 h in Krebs Ringer Hepes buffer (KRH buffer: 130 mM NaCl, 5 mM KCl, 1.3 mM CaCl_2_, 25 mM HEPES, 10 mM Na_2_HPO_4_, 1.3 mM MgSO_4_, 0.2% BSA), in the presence or absence of the different treatments: glucose (200 mM), or glutamine (10 mM), or ABA (0.1, 10 or 200 μM).

After treatments, medium and cells were collected separately: GLP-1 content in the supernatant was analyzed by GLP-1 Total ELISA Kit (Merck Millipore, Vimodrone, MI, Italy); total protein content in cells was analyzed by Bradford assay (Bio-Rad, Milano, Italy).

### Quantitative real time-PCR

Total mRNA was extracted from hNCI-H716 using Qiazol (Qiagen, Milan, Italy) according to the manufacturer's instructions. Quality and quantity of RNA were analysed using a NanoDrop spectrophotometer (Nanodrop Technologies, Wilmington, DE). The cDNA was synthesized by the iScriptTM cDNA Synthesis Kit (Bio-Rad, Milan, Italy) starting from 1 μg of total RNA. PCR primers were designed through Beacon Designer 2.0 Software and their sequences were as indicated in Table 1.

**Table 1:** Primers.

Human gene		Sequence, 5’-3’
**GLP-1**	Forward	GCTGAAGGGACCTTTACCAGT
	Reverse	CCTTTCACCAGCCAAGCATG
**GLUCAGON**	Forward	ATTCACAGGGCACATTCACCA
	Reverse	GGTATTCATCAACCACTGCAC
**ACTIN**	Forward	GCGAGAAGATGACCCAGATC
	Reverse	GGATAGCACAGCCTGGATAG
**HPRT-1**	Forward	GGTCAGGCAGTATAATCCAAAG
	Reverse	TTCATTATAGTCAAGGGCATATCC

qPCR was performed in an iQ5 real-time PCR detection system (Bio-Rad) using 2× iQ Custom Sybr Green Supermix (Bio-Rad). Values were normalized on mRNA expression of human β-actin and HPRT. Statistical analysis of the qPCR was performed using the iQ5 Optical System Software version 1.0 (Bio-Rad) based on the ^2−^ΔCt method [7]. The dissociation curve for each amplification was analysed to confirm absence of unspecific PCR products. Experiments were repeated three times in triplicate.

### Measurement of the intracellular cAMP concentration

hNCI-H716 cells were seeded at the density of 5x10^5^/well in 12-well, Matrigel matrix-coated plates. After 24 h, cells were washed with HBSS, pre-incubated for 10 min in HBSS containing 10 μM IBMX, an inhibitor of phosphodiesterases, and then stimulated with 10 mM glutamine or 200 μM ABA for 2.5 and 5 min.

Supernatant was removed and cells were lysed in 0.6 M PCA. Intracellular cAMP content was evaluated by EIA (Cayman, Ann Arbor, MI, USA) on neutralized extracts [18].

### Vector construction

The full length LANCL2 cDNA was amplified by PCR using cDNA obtained with reverse transcription of total RNA from human granulocytes and using the following primers: 5’-CACCATGGGCGAGACCATGTCAAAG-AG-3’(foward); 5’-ATCCCTCTTCGAAGAGTCAAGTTC-3’ (reverse).

The PCR was performed in 25 μl containing undiluted reaction buffer, 200 μM dNTP, 5 pmol of primers and using 1.25 U of Herculase HotStart DNA polymerase. The PCR reaction profile was 1 cycle at 94°C for 2 min, 35 cycles at 94°C for 15 s, 62°C for 30 s and 72°C for 1 min with a final extension for 5 min at 72°C. The PCR product was purified with Nucleospin^®^ Extract Kit (Macherey-Nagel) and cloned into pcDNA3.1/V5-His-TOPO^©^. This vector allows the synthesis of the recombinant protein as a C-terminal fusion to the V5 epitope and a Histidine tag. The LANCL2 plasmid was purified using PureLink™ HiPure Plasmid Filter Kit (Invitrogen) and sequenced by TibMolbiol (Genova, Italy).

### LANCL2 overexpression

hNCI-H716 cells were transfected in parallel with pcDNA3.1(+) (control plasmid) or with the plasmid containing the full-length LANCL2 cDNA, LANCL2-pcDNA3.1(+) (LANCL2 plasmid). Transient transfection of hNCI-H716 cells (1.5x10^6^) was performed using the Nucleofector System (Amaxa GmbH, Köln, Germany), program X-005, solution T, with 3 μg LANCL2-plasmid or control plasmid. hNCI-H716 cells were then resuspended in DMEM and seeded in Matrigel-coated 24-well plates. Experiments were performed 48 h after transfection.

### Western blot analysis

hNCI-H716 cells (2.5x10^5^) were lysed in 50 μl HES lysis buffer (20 mM Hepes, pH 7.4, 1 mM EDTA, 250 mM sucrose) containing a protease inhibitor cocktail (Sigma), and LANCL2 expression was analyzed by Western blot, using a monoclonal antibody against LANCL2 [19]. LANCL2 expression was normalized on vinculin levels, detected with a goat polyclonal antibody against actin (Santa Cruz Biotechnology, Dallas, TX). Appropriate HRP-conjugated secondary antibodies (Cell Signaling, Danvers, MA) and enhanced chemiluminescence reagents (GE Healthcare, Little Chalfont, Buckinghamshire, UK) were used to detect antigens after transfer to a nitrocellulose membrane.

### In vivo experiments

Two-months old female Wistar rats weighing 160 to 198 g (obtained from Charles River Laboratories Italia, Calco, LC, Italy) were housed singly under a 12 h/12 h light/dark cycle under free feeding conditions, in temperature- and humidity-controlled rooms.

After an overnight fast, the DPP4 inhibitor Sitagliptin (Januvia^®^, 10 mg/Kg) [20], was orally administered 30 min prior to ABA (50 mg/Kg) or vehicle (water) gavage. After anesthesia with ketamine/xylazine, blood samples were collected at 0, 20, 40 and 60 min by orbital sinus bleeding in heparin and plasma aliquots were stored at -20°C.

The dose of ABA was chosen based on the effect of dietary ABA supplementation [13].

In other experiments, where animals were not pre-treated with Sitagliptin, GLP-1 concentration was also evaluated in the portal vein blood, as in [21], 10 min after intragastric vehicle or ABA administration.

### Measurements of plasma GLP-1, glucose, insulin and ABA

GLP-1 concentrations were determined by ELISA (Merck Millipore; the kit detects the total GLP-1 levels). Glycemia was measured with a glucometer (Bayer, Milano, Italy) and insulinemia by ELISA (Bertin-Pharma, Montigny, France). ABA plasma concentrations were determined by ELISA, as in [5].

### Ethics statement

Animal rearing conditions were consistent with the guidelines of the Italian Ministry of Health and the study was approved by the IRCCS AOU San Martino-IST Ethical Committee (Genova, Italy).

## Results

### ABA stimulates GLP-1 secretion

**Fig 1:**
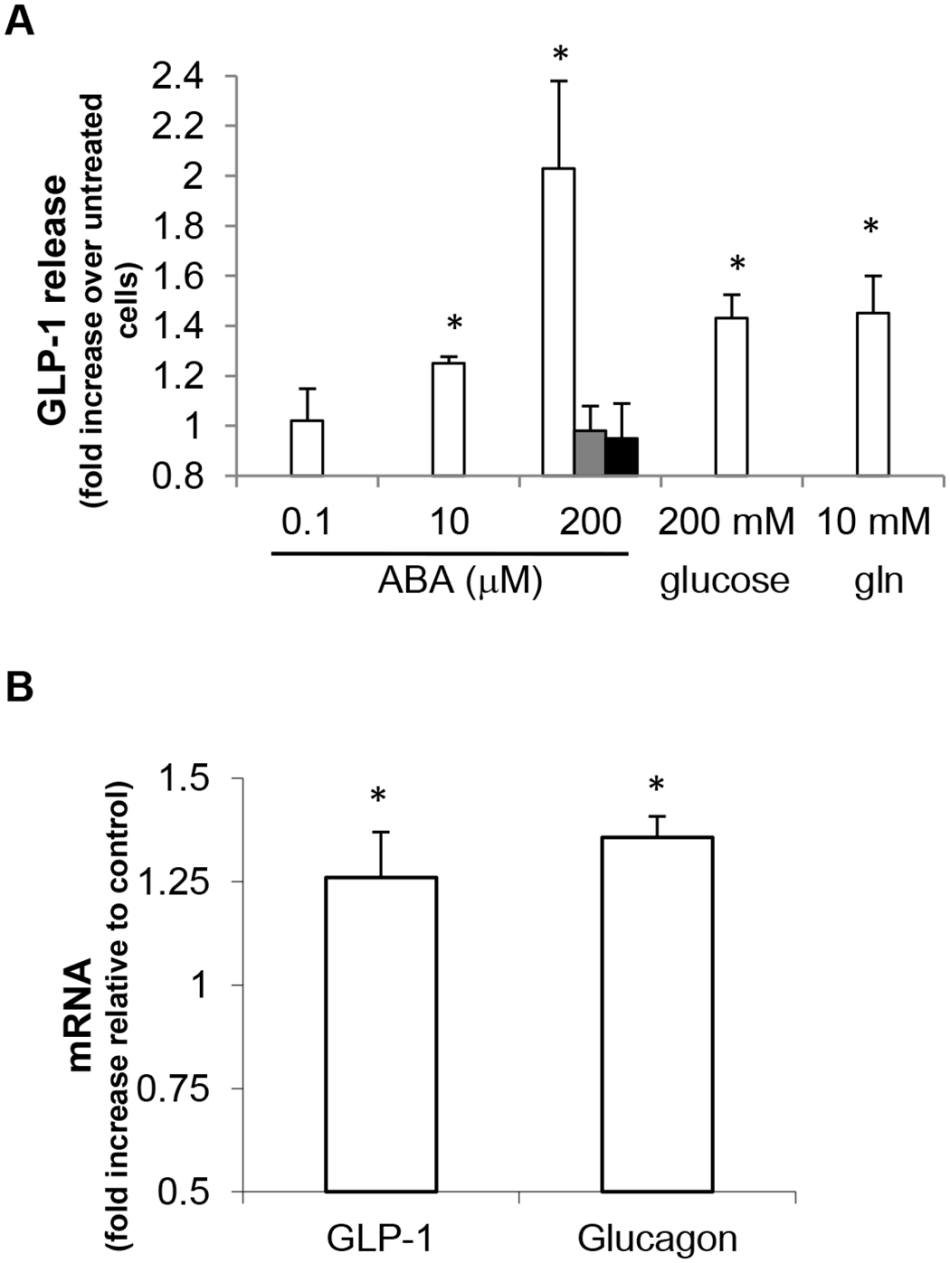
ABA induces GLP-1 release and transcription in hNCI-H716 cells. (A) hNCI-H716 cells were incubated for 2 h in the absence or presence of ABA (at the indicated concentrations), or of 200 mM glucose or 10 mM glutamine (gln). In some experiments, cells were pre-incubated for 10 min in the absence or presence of 20 μM 2′,3′-Dideoxyadenosine, a specific adenylyl cyclase inhibitor (grey bar) or of 1 μM of a cell permeable PKA inhibitor (protein kinase A inhibitor 14–22 amide, myristoylated, black bar), prior to stimulation with 200 μM ABA. GLP-1 levels in the culture media were then estimated with an ELISA kit. Data, expressed as fold increase over values in untreated cells, are expressed as mean±SD of at least 3 different experiments. *, p<0.05 compared to untreated cells. (B) hNCI-H716 cells were incubated for 2 h in the absence or presence of 200 μM ABA and qPCR was performed with specific primers for GLP-1 and glucagon; *, p<0.05 compared to expression in untreated cells.

hNCI-H716 cells were challenged for 2 h with different ABA concentrations and GLP-1 levels were measured in the supernatants. The basal GLP-1 concentration was 347±106 pM. As shown in Fig 1A, 200 μM ABA approximately doubled the extent of the GLP-1 secretion. 10 μM ABA was sufficient to trigger a statistically significantly higher GLP-1 release, compared to the untreated control. No stimulation of GLP-1 secretion was obtained in the presence of 100 nM ABA. The calculated EC50 for the ABA-induced GLP-1 release was 23±3 μM (not shown). To compare the effect of ABA on GLP-1 release with that of other secretagogues, cells were also incubated in the presence of 200 mM glucose or 10 mM glutamine [22, 23]: GLP-1 secretion was increased by approximately 1.4-fold with both stimuli (Fig 1A).

Interestingly, ABA treatment also significantly increased preproglucagon mRNA levels, as demonstrated by qPCR using two different sets of primers, specific for GLP-1 and glucagon, respectively, yielding a similar result (Fig 1B).

### The ABA-induced GLP-1 secretion is mediated by a cAMP-dependent mechanism

**Fig 2:**
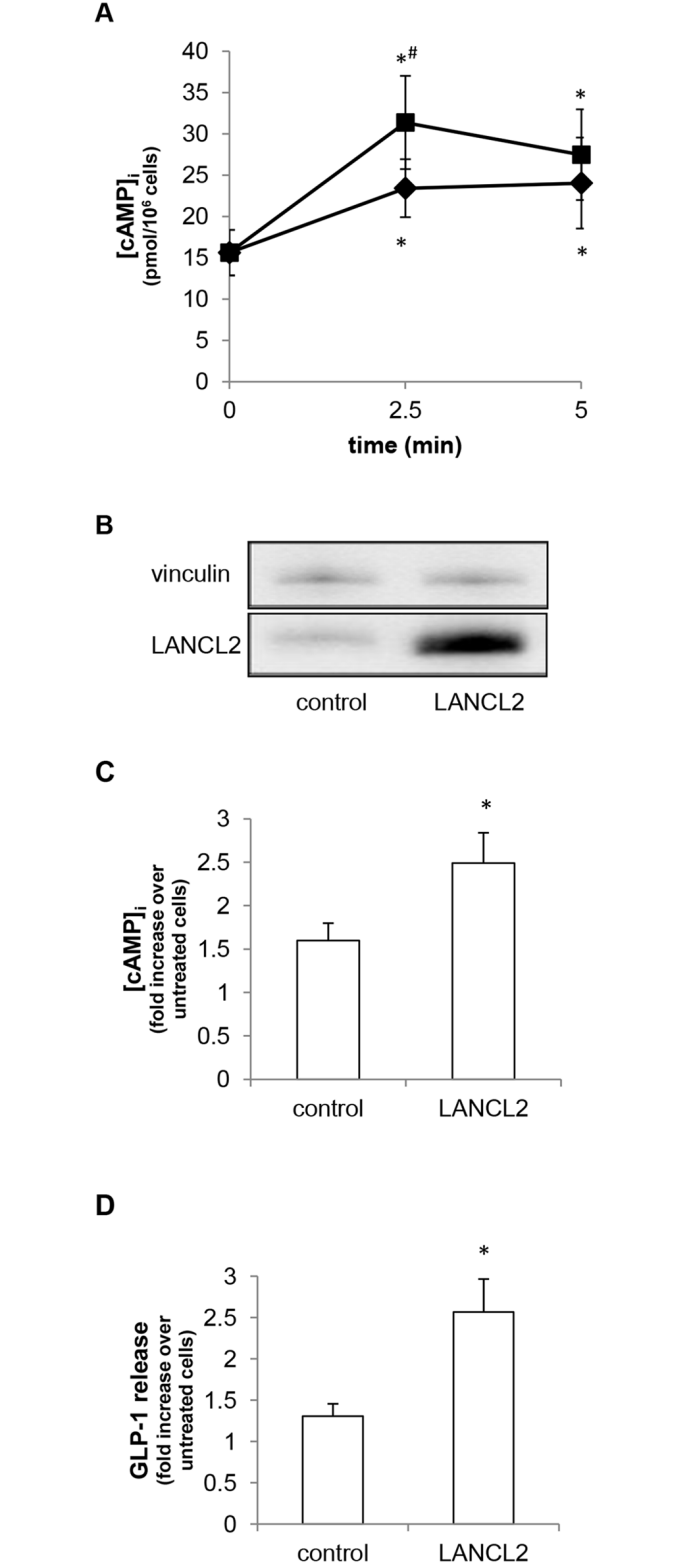
ABA induces the increase of the [cAMP]_i_ in hNCI-H716 cells. (A) hNCI-H716 cells were incubated for the indicated time in the absence or presence of 200 μM ABA (squares), or of 10 mM glutamine (rhombi); [cAMP]_i_ was then measured on cell extracts. Data are mean±SD of at least 3 different experiments; *, p<0.05 compared with untreated cells; #, p<0.05 compared to glutamine-treated cells (for the same time). (B) hNCI-H716 cells were transfected with an empty plasmid (control) or with a LANCL2-containing plasmid (LANCL2). After 48 h from transfection, cells were lysed and a Western blot analysis was performed using an anti-LANCL2 monoclonal antibody [19]; a representative blot is shown, confirming LANCL2 overexpression after transfection. LANCL2 expression was normalized on vinculin levels. (C) After 48 h from transfection, cells were stimulated for 2.5 min in the absence or presence of 200 μM ABA. [cAMP]_i_ was measured on cell extracts and data, expressed as fold increase over values in unstimulated cells, are expressed as mean±SD of at least 3 different experiments; basal cAMP values were not significantly different upon transfection. *, p<0.05 compared to control. (D) After 48 h from transfection, cells were incubated for 2 h in the absence or presence of 200 μM ABA. GLP-1 levels in the culture media were then estimated with an ELISA kit. Data, expressed as fold increase over values in unstimulated cells, are expressed as mean±SD of at least 3 different experiments. *, p<0.05 compared to untreated cells.

In different human cell types, the cell-specific ABA-induced response is mediated by an increase of the second messenger cAMP [3, 4, 7, 9, 24], and by the consequent PKA activation [3, 4, 25]. Since GLP-1 release is regulated by the [cAMP]_i_ [14], we verified whether ABA was able to induce an increase of the [cAMP]_i_ in hNCI-H716 cells. As a positive control, cells were incubated with glutamine, which is known to determine a [cAMP]_i_ increase in hNCI-H716 cells [26]. As shown in Fig 2A, a 2.5-min incubation in the presence of 200 μM ABA induced a 2-fold increase of the [cAMP]_i_, while 10 mM glutamine increased the [cAMP]_i_ approximately 1.4-fold.

In mammalian cells, the ABA-induced cAMP increase is mediated by the protein LANCL2 [7, 9]. hNCI-H716 cells were transfected by electroporation with an empty plasmid, or with a plasmid containing the full-length cDNA for human LANCL2. LANCL2 overexpression, confirmed by Western blot analysis with a specific monoclonal antibody (Fig 2B), was accompanied by a significant increase in ABA-induced cAMP accumulation, as compared to cells transfected with an empty plasmid (Fig 2C), as well as by a significant increase in ABA-induced GLP-1 release (Fig 2D). The ABA-induced [cAMP]_i_ increase and GLP-1 release were approximately 1.4-fold in cells transfected with the empty plasmid (control bars in Fig 2D), and not 2-fold as observed in untransfected cells (Figs 1A and 2A), indicating that cell responsiveness was slightly affected by the transfection procedure *per se*.

In order to verify whether the ABA-induced [cAMP]_i_ increase mediates the ABA-stimulated GLP-1 release, hNCI-H716 cells were pre-incubated in the presence of a specific adenylyl cyclase inhibitor (2′,3′-Dideoxyadenosine), or a cell permeable PKA inhibitor: both inhibitors abrogated the GLP-1 release stimulated by 200 μM ABA (Fig 1A).

### ABA increases plasma GLP-1 in rats

**Fig 3:**
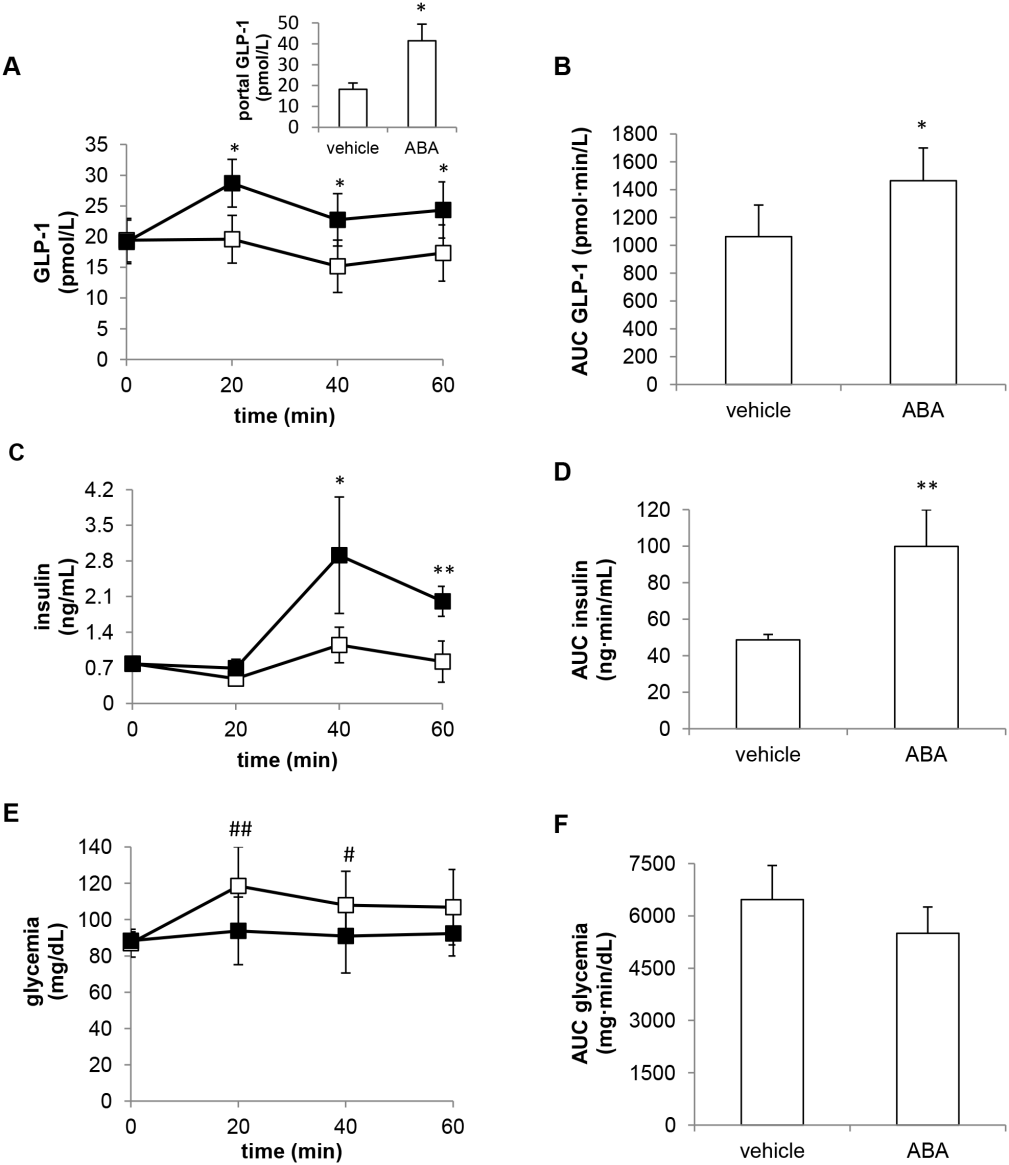
Effect of oral ABA on plasma GLP-1, insulin and glucose levels in rats. ABA (50 mg/Kg, black squares) or vehicle alone (open squares) were orally administered to rats pre-treated with Sitagliptin (6 animals per experimental group) and blood samples were collected at 0, 20, 40 and 60 min to evaluate plasma GLP-1 (A), insulin (C) and glucose (E). The AUC corresponding to the curves of GLP-1 (B), insulin (D) and glycemia (F) were calculated. Inset to panel A: blood samples were collected from the portal vein of rats not pre-treated with Sitagliptin, 10 min after ABA or vehicle administration and GLP-1 levels were evaluated (n = 5 rats per group). *, p<0.05 and **, p<0.01 compared with the corresponding value in vehicle-treated animals; #, p<0.05 and ##, p<0.01 compared with time zero.

First, we examined the effect of a single-dose oral administration of ABA (at 50 mg/Kg) on GLP-1 levels in normal rats (6 animals per experimental group) pre-treated with Sitagliptin. 20 min after ABA administration, plasma GLP-1 (GLP-1p) increased by approximately 50%, whereas the vehicle alone had no effect on GLP-1p levels (Fig 3A). The area under the curve of GLP-1p (GLP-1p AUC) over the entire time frame was calculated from GLP-1p values relative to time zero: the GLP-1p AUC was significantly higher in the ABA-treated compared to the control animals (Fig 3B).

GLP-1 levels also significantly increased in the portal vein blood of rats not pre-treated with Sitagliptin 10 min after ABA administration (Fig 3A, inset), indicating that ABA alone is capable of increasing plasma GLP-1. ABA concentration in the portal vein blood was in the low nM (4.2±1.9 nM) range in the vehicle-treated animals and in the μM range (3.9±0.4 μM) in the ABA-treated animals.

The observation that ABA induced an increase of GLP-1p, together with the fact that exogenous ABA is known to directly stimulate insulin release from β-cells *in vitro* [4], prompted us to measure insulin levels in the ABA-treated rats. As shown in Fig 3C and 3D, insulinemia indeed significantly increased after ABA administration and the plasma insulin AUC was consequently higher in the ABA-treated than in the vehicle-treated group.

Glycemia was slightly increased in vehicle-treated animals: this increase was not observed upon oral ABA administration (Fig 3E and 3F). The increase of glycemia observed in the control animals can be attributed to anesthesia: indeed, ketamine/xylazine have been shown to induce hyperglycemia in fed rats and, to a lower extent, also in fasted animals [27], as in our experimental protocol.

In conscious rats, oral ABA administration at the same dose used in the anesthetized animals (50 mg/Kg) resulted in a slight, yet significant reduction of blood glucose after 60 min (81±6 mg/dL, n = 6) compared with time zero values (92±10, n = 12, p = 0.03) and with values measured at the same time point in the vehicle-treated controls (99±14, n = 6; p = 0.02).

## Discussion

We had previously demonstrated that GLP-1 stimulates ABA release from β-pancreatic cells [5]. In this study, we show that ABA can induce GLP-1 release, indicating a positive feed-back between these two molecules, possibly relevant to glycemia regulation.

The molecular mechanism by which ABA induces GLP-1 release by hNCI-H716 cells is similar to the one described in several other cell systems (including immune cells, β-pancreatic cells and endothelial cells), i.e. through the cAMP/PKA axis [3, 4, 6, 7, 9, 24, 25]. Indeed, this signaling pathway is known to regulate GLP-1 release also in response to other stimuli, such as glucose and glutamine [14, 15, 26].

In the *in vivo* experiments, we chose to administer a dose of ABA of 50 mg/Kg, based on the results obtained by Guri et al. [13], showing that dietary ABA at 100 mg/Kg, introduced over a 24-h period, was effective in reducing glycemia in db/db mice fed a high-fat diet. We hypothesized that a smaller dose could also be effective, if bolus-administered by gavage.

The plasma insulin increase upon ABA administration was expected, based on the *in vitro* effect of ABA on insulinoma cells and on murine and human β-cells: exogenous ABA, added at concentrations in the low nM range, stimulated insulin secretion both in the presence and absence of glucose [4]. The plasma ABA concentration measured in the rats 10 min after oral ABA administration was in the micromolar range and could thus be responsible for the observed increase of plasma insulin (Fig 3C). The increase of plasma GLP-1 peaked at 20 min (Fig 3A), preceding the insulin increase (which conversely was maximal at 40 min, Fig 3C). This timing of events suggests that ABA stimulated the release of both GLP-1 and insulin. Since GLP-1 can stimulate ABA release from β-cells *in vitro*, both in the presence or absence of glucose [5], one might envisage a feed-back mechanism whereby GLP-1 contributes to stimulate endogenous ABA release, which in turn further stimulates insulin secretion.

In control animals, glycemia was significantly higher at both 20 and 40 min compared with time zero. The increase of glycemia observed in the control animals might be attributed to anesthesia, as described by Saha JK et al [27], who showed that ketamine and xylazine significantly altered glycemia in fed and, to a much lower extent, also in fasted rats, as occurred in our study (Fig 3E, white squares).

In ABA-treated rats, the increase of glycemia was not observed, neither at 20 nor at 40 min (Fig 3E, black squares): while, at 40 min, a significant increase of insulinemia (Fig 3C) may have contributed to glycemia control, at 20 min insulinemia was not (yet) increased. Thus, two mechanisms may be responsible for the maintenance of normal blood glucose levels in the ABA-treated, anesthetized animals: i) an “early” (0–20 min), insulin-independent glycemia lowering effect of ABA, followed by, ii) a “late” (20–40 min) glycemia reducing effect, attributable to the increase of insulinemia. We previously reported that ABA can trigger glucose uptake in myocytes and in adipocytes [5], to a similar extent as that observed with insulin at the same concentration (i.e. 100 nM). Thus, the normalization of glycemia observed in the ABA-treated animals compared with the controls could result from stimulation by ABA of both glucose transport and insulin release. The plasma concentration of ABA measured 10 min after its administration, which was in the micromolar range, was markedly higher than the one capable of stimulating myoblast glucose uptake *in vitro* [5].

Type 2 diabetes treatments targeting the GLP-1 axis by either inhibiting its clearance by DPP4 or using GLP-1 mimetics [15] are currently used. More recently, treatments aimed at stimulating GLP-1 release from L cells have been considered as an alternative approach and our finding that ABA increases GLP-1 release may indicate ABA and/or ABA analogs activating LANCL2 as potential anti-diabetic treatments, alone or in combination with DPP4 inhibitors. Indeed, detection of high GLP-1 levels in the portal vein upon ABA administration demonstrates that oral ABA alone is capable of increasing plasma GLP-1 (Fig 3A). Identifying LANCL2-activating compounds might indeed prove a successful strategy, in view of the multiple anti-diabetic effect that they might trigger. Indeed LANCL2 can: i) mediate the ABA-induced insulin release [7]; ii) facilitate Akt phosphorylation [28], and increase GLUT4-mediated glucose uptake [5], which is an Akt-dependent mechanism [29]; iii) mediate the ABA-stimulated GLP-1 release from L cells (Fig 2D). The potential role of LANCL2 as a new drug target in diabetes has been also suggested by other authors [30] and efforts at discovering LANCL2-targeting drugs have been reported [31, 32].

Besides its effects on glycemia regulation, GLP-1 also exerts protective effects on the cardiovascular system [14]: thus, ABA administration, resulting in GLP-1 release, might be beneficial also in this respect, together with improving glycemic homeostasis. Indeed, ABA administration has been reported to improve atherosclerosis in ApoE^-/-^ mice [24].

So far, it is not possible to exclude that, when administered *in vivo*, ABA can also trigger GLP-1 release from organs/tissues other than L-type cells, e.g. α-pancreatic cells [15]. Moreover, it remains to be defined whether ABA stimulates GLP-1 release acting on the intestinal lumenal side of L-cells, like a nutrient, or whether plasma ABA can stimulate GLP-1 secretion also acting as a hormonal stimulus on the vascular side of L-cells, as happens for glucose [14]. Indeed, GLP-1 release is evoked in response to multiple paracrine, neural and hormonal stimuli [14, 15]. Finally, future studies should explore the effect on GLP-1 release by ABA administered together with nutrients.

## Abbreviations

ABA: abscisic acid
[ABA]_p_: plasma ABA concentration
LANCL2: lanthionine synthetase C-like protein 2
GLP-1: glucagon-like peptide 1
FBS: fetal bovine serum
HBSS: Hank’s Balanced Salt Solution
KRH: Krebs Ringer buffer
PCA: perchloric acid
qPCR: quantitative real time PCR
GLP-1p: plasma GLP-1
AUC: area under the curve
DPP4: dipeptidyl-peptidase IV
